# Epidermal Growth Factor Receptor Expression and Resistance Patterns to Targeted Therapy in Non-Small Cell Lung Cancer: A Review

**DOI:** 10.3390/cells10051206

**Published:** 2021-05-14

**Authors:** Emma-Anne Karlsen, Sam Kahler, Joan Tefay, Shannon R. Joseph, Fiona Simpson

**Affiliations:** 1 Simpson Laboratory, The University of Queensland Diamantina Institute, Woolloongabba, Brisbane 4102, Australia; s.joseph@uq.edu.au (S.R.J.); f.simpson@uq.edu.au (F.S.); 2 Department of General Surgery, Mater Hospital Brisbane, South Brisbane 4101, Australia; 3 Faculty of Medicine, The University of Queensland, St Lucia 4067, Australia; samkahler96@gmail.com (S.K.); joan.tefay@gmail.com (J.T.); 4 Department of General Surgery, Redland Hospital, Cleveland 4163, Australia

**Keywords:** epidermal growth factor receptor, non-small cell lung cancer, drug resistance, tyrosine kinase inhibitor, monoclonal antibody

## Abstract

Globally, lung cancer is the leading cause of cancer-related death. The majority of non-small cell lung cancer (NSCLC) tumours express epidermal growth factor receptor (EGFR), which allows for precise and targeted therapy in these patients. The dysregulation of EGFR in solid epithelial cancers has two distinct mechanisms: either a kinase-activating mutation in EGFR (EGFR-mutant) and/or an overexpression of wild-type EGFR (wt-EGFR). The underlying mechanism of EGFR dysregulation influences the efficacy of anti-EGFR therapy as well as the nature of resistance patterns and secondary mutations. This review will critically analyse the mechanisms of EGFR expression in NSCLC, its relevance to currently approved targeted treatment options, and the complex nature of secondary mutations and intrinsic and acquired resistance patterns in NSCLC.

## 1. Introduction

Lung cancer is the leading cause of cancer-related mortality amongst both men and women, accounting for approximately 25% of all cancer deaths globally [1]. There are two main forms of lung cancer: non-small cell lung cancer (NSCLC) and small cell lung cancer (SCLC), which account for 85% and 15% of diagnoses, respectively [2]. The World Health Organisation has subclassified NSCLC into three main types: adenocarcinoma, squamous cell carcinoma, and large cell [3,4]. Adenocarcinoma is the most common type of NSCLC, accounting for approximately 40% of lung cancers [3]. Although tobacco smoking is a major risk factor for lung cancer, approximately 10–25% of all lung cancers occur in nonsmokers [5]. In non-smokers, carcinogenesis is linked to the presence of distinct oncogenic driver mutations, including epidermal growth factor receptor (EGFR), proto-oncogene B-Raf (BRAF), MET proto-oncogene (MET), and Kirsten rat sarcoma viral oncogene (KRAS) [6].

EGFR was the first oncogenic target discovered in NSCLC and is present in over 60% of patients [7,8,9]. The presence of activating EGFR mutations is associated with female gender and adenocarcinoma histology and inversely associated with smoking history [10]. EGFR is a commonly altered oncogene in other solid epithelial cancers, such as colorectal cancer, head and neck cancer, pancreatic cancer, breast cancer, and glioblastoma [11]. However, two distinguishable mechanisms exist between these cancers: either a kinase-activating mutation in EGFR (EGFR-mutant) or an overexpression of the EGFR protein (wt-EGFR). Kinase-activating mutations lead to increased tyrosine kinase activity in EGFR and are frequently present in NSCLC and glioblastoma but rarely in other types of cancers (Table 1) [7,8,12,13,14]. Overexpression of wt-EGFR protein can be present with or without underlying EGFR gene amplifications and is often associated with a negative prognosis [15,16,17,18,19,20,21,22]. The underlying mechanism behind EGFR-positive cancers, whether it be mutations, gene amplification, or overexpression, has a significant impact on the efficacy of anti-EGFR therapies and resistance patterns.

## 2. EGFR Molecular Biology

### Receptor Structure and Activation

The EGFR belongs to the ErbB family of tyrosine kinase receptors, which comprises four family members: EGFR/HER1/erbB1, HER2/erbB2/neu, HER3/erb3, and HER4/erb4 [23]. Each family member has an extracellular domain (ECD) with two cysteine-rich regions, a single transmembrane or membrane-spanning region, a juxtamembrane cytoplasmic domain, and an intracellular kinase (or pseudokinase domain) with multiple C-terminal tyrosine residues that are phosphorylated on ligand binding and receptor activation [24,25]. There are crucial structural and functional differences between the ErbB receptor family members. In particular, ErbB3 lacks kinase activity due to the substitution of amino acids in the kinase domain, while ErbB2 has no known ligand binding capacity [26]. This has significant implications in receptor activation and subsequent intracellular signalling.

Growth factors that bind to ErbB receptors belong to the EGF-family and are typically secreted in an autocrine and paracrine fashion [27]. The binding of growth factors to ErbB receptors induces dimerisation via interactions between the extracellular region [27]. Although all forms of dimerisation between the ErbB receptors are possible, not all are biologically significant [27]. Ligand binding to EGFR induces homodimerisation, as well as heterodimerisation with ErbB2, ErbB3 and ErbB4 receptors [28]. ErbB2 receptor is only activated when heterodimerisation occurs with another ErbB receptor [24]. Heterodimerisation also serves to increase the repertoire of intracellular signalling pathways utilised for downstream effects [28].

**Figure 1:**
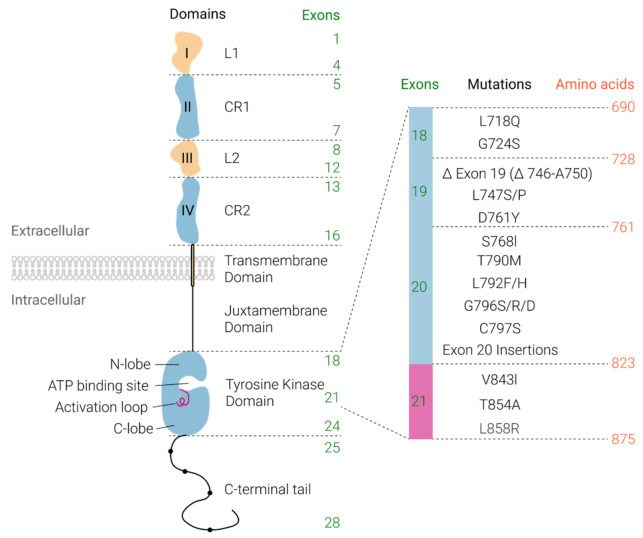
A schematic representation of EGFR illustrating exon boundaries and associated extracellular, transmembrane, and intracellular protein domains. The extracellular domain is involved in ligand binding (Domains I and III) and dimerisation (Domain II). The intracellular domain contains a juxtamembrane domain, tyrosine kinase domain, and multiple C-terminal tyrosine residues (circles), which are phosphorylated on ligand binding and receptor activation leading to the activation of cell proliferation, survival, migration, and/or angiogenesis signalling pathways. The tyrosine kinase domain is expanded to show relevant mutations associated with resistance and sensitivity to first-, second- and third-generation tyrosine kinase inhibitors as discussed in Table 3. (large EGF binding domain 1, L1; cysteine-rich domain 1, CR1; large EGF binding domain 2, L2; and cysteine-rich domain 1, CR2).

EGFR is located on chromosome 7 short arm q22 [28]. Mature EGFR contains 1186 residues and is formed when a 1210 residue-containing precursor is cleaved at the N-terminal [29]. The extracellular domain of EGFR is made of 621 amino acids and comprises four subdomains, I (large EGF binding domain 1 (L1), amino acids 1–333, exons 1–4), II (cysteine-rich domain 1 (CR1), amino acids 134–152, exons 5–7), III (large EGF binding domain 2 (L2), amino acids 313–445, exons 8–12), and IV (cysteine-rich domain 2 (CR2), amino acids 446–621, exons 13–16) [26,29] (Figure 1). The transmembrane region is a single 23-amino acid long hydrophobic α-helical peptide. The tyrosine kinase domain is made of conserved amino acids 690–953 (exons 18–24) and is divided into an N-lobe and a C-lobe (Figure 1). Between the two lobes lies the adenosine triphosphate (ATP)-binding site for phosphorylation [26,29]. Finally, the C-terminal fragment rich in tyrosine residues (amino acids 954–1136, exons 25–28) enables coupling with intracellular signalling proteins [29].

Six ligands (EGF, transforming growth factor alpha (TGFα), amphiregulin, betacellulin, heregulin, and heparin-binding EGF) are known to bind to EGFR. [30]. These ligands interact with two (I and III) of four subdomains rich in cysteine residues that comprise the extracellular domain of ErbB receptors [27,28]. Subdomain II contains a beta hairpin (also called a dimerisation loop) that facilitates homo/heterodimerisation. This brings into proximity the cytoplasmic kinase domains, which facilitate the activation of the cytoplasmic component through both phosphorylation-dependent and nondependent mechanisms [23,31]. This subsequently enables coupling with intracellular downstream signalling molecules containing Src homology 2 (SH2) and phosphotyrosine binding (PTB) domains [27,28].

Three main signalling pathways are activated downstream of EGFR: the Ras/Raf/Mitogen-activated protein kinases (MAPK) pathway, the phosphatidylinositol 3-kinase (PI3K)/AKT8 virus oncogene cellular homolog the (AKT)/Mammalian target of rapamycin (mTOR) pathway and the signal transducer and activator of the transcription (STAT) pathway [9,28]. In the first pathway, adaptor protein Grb2 containing SH2 domain binds to activated EGFR and interacts with and activates Ras [28]. This in turn activates Raf-1, and the downstream pathway leads to nuclear transcription factor activation via extracellular signal-regulated kinases (ERK)-1 and ERK-2 MAP kinases [28]. The Ras/Raf/MAPK pathway is involved in cell proliferation, survival, migration, and angiogenesis.

The PI3K/AKT/mTOR pathway is involved in cellular metabolism and motility [28,29]. PI3K interacts with EGFR via the adaptor protein Grb2-associated binding protein 1 (GAB1) and Ras. Activated PI3K recruits and activates AKT. Activated AKT regulates cell death in an antiapoptotic manner and also signals mTOR, which directly regulates cellular metabolism and cell growth [29].

The STAT pathway was first identified as part of the intracellular signalling pathway of cytokine receptors [28,30]. STAT binds to EGFR via the SH2 domain and is phosphorylated, leading to dimerisation. Activated STAT complexes translocate to the nucleus to regulate the transcription of genes involved in cellular proliferation, cell cycle progression, and apoptosis [30].

There are three main mechanisms leading to EGFR activation: increased EGFR expression in malignant cells, increased ligand production, and the presence of EGFR-activating mutations (Table 1) [32]. Although EGFR overexpression was originally thought to be a promising therapeutic focus, the specific targeting of activating mutations in cancers such as NSCLC has emerged as a superior therapeutic strategy.

## 3. EGFR in NSCLC

Dysregulated EGFR signalling is a well-established phenotype in NSCLC [26]. It can be caused by either overexpression or activating mutations [26]. Overexpression of EGFR is found to occur in approximately 60% of NSCLCs [26]. Additionally, molecular profiling studies have shown activating mutations in EGFR occur in 10–15% of Caucasian patients and at least 50% of Asian patients with NSCLC [71,72,73]. The evidence for a correlation between EGFR gene amplification and protein expression is conflicting, with several studies providing evidence both for and against the association [38,74,75,76,77].

The “classical” EGFR mutations consist of a deletion in exon 19 and a single amino acid substitution L858R in exon 21 and account for 47% and 41% of the EGFR mutations in NSCLC, respectively [78]. Rare mutations account for the remaining 12% and consist of point mutations, deletions, and insertions within exons 18–25 of the EGFR gene [79]. In general, these oncogenic mutations lead to a constitutive activation of the tyrosine kinase domain of EGFR (amino acids 690–952, exons 18–24) [29].

## 4. EGFR-Targeted Therapy in NSCLC and Mechanisms of Resistance

Anti-EGFR therapies consist of either monoclonal antibodies (mAbs) that are directed at the extracellular portion of the EGFR or small molecule tyrosine kinase inhibitors (TKIs) that target the intracellular protein kinase domain. Table 2 summarises the currently approved anti-EGFR therapies and their respective indications.

### 4.1. Tyrosine Kinase Inhibitors (TKIs)

TKIs inhibit receptor signalling by competitively blocking the binding of ATP to the cytoplasmic domain of the EGFR. These medications are associated with a high objective response rate, ranging from 50–77% [80,81,82,83]. In original studies, it was noted that certain characteristics, such as adenocarcinoma histology, Asian ethnicity, and minimal smoking history, were correlated with improved response to TKI therapy [71,84,85,86,87,88,89,90,91]. However, subsequent molecular testing of patients who had responded to TKIs demonstrated that somatic activating mutations in EGFR, were in fact underpinning the response [33,86,87,92,93].

The best characterised mutations that confer sensitivity to EGFR-TKI therapy are located in exon 19 (deletions, especially E746-A750del) and exon 21 (L858R) [92]. These classical mutations were investigated by Lynch et al. in 2004, who reported that EGFR mutations were related to the sensitivity of NSCLC to gefitinib, a first-generation TKI [93]. This work was supported by Paez et al. who demonstrated that these EGFR mutations were correlated with a clinical response to gefitinib [94]. EGFR TKIs are currently approved for the treatment of metastatic NSCLC with classical mutations (Table 2). Uncommon but pertinent EGFR mutations that confer sensitivity to TKI therapy include exon 18 point mutations (G719S/A/C) and exon 20 insertions (A763_Y64insFQEA) [92].

In patients with EGFR-mutant NSCLC, the Riesa Pan-Asia Study (IPASS) was the first to demonstrate a significant improvement in 12-month progression-free survival (PFS) with gefitinib, 24.9%, compared to chemotherapy, 6.7% [95]. In contrast, patients with wt-EGFR had better outcomes with chemotherapy. This trial was subsequently supported by the First-SIGNAL study which demonstrated that, in patients treated with gefitinib, an EGFR-activating mutation predicted to have superior overall response rate (ORR) (84.6% vs. 37.5%) [96]. Several trials have compared the utility of gefitinib, erlotinib, or afatinib to chemotherapy specifically in patients with EGFR-mutant NSCLC [80,81,82,97,98,99]. All have demonstrated that first-line EGFR TKIs resulted in improved ORR, PFS, and quality of life compared to chemotherapy. This has formed the basis of TKIs being used as a first-line therapy in patients with EGFR-activating mutations.

The role of TKIs in patients with wt-EGFR NSCLC is controversial. Due to the results of the IPASS and TORCH trials, which demonstrated inferior survival compared to chemotherapy, first-line TKI therapy is not recommended in this population [84,95,100,101,102,103]. The TAILOR trial compared erlotinib to docetaxel specifically in wt-EGFR tumours and demonstrated significantly poorer ORR, PFS, and overall survival with the anti-EGFR therapy [104].

Consecutive analysis revealed that while 75% of patients with the common TKI-sensitive L858R or 19del mutations respond to first-generation TKIs, an acquired resistance developed that limited PFS to less than 12 months [105,106,107,108,109,110,111,112,113,114]. The third-generation TKI, osimertinib, was developed to address the most common T790M-mediated resistance mechanism yet stable disease was limited to a PFS of 9.9 months [115]. Progression is understood to arise from clones with secondary EGFR mutations, enhanced signalling in a downstream or alternate pathway, histologic transformation, or mechanisms of transcriptional regulation (Table 3). Advances in understanding of these pathways and techniques to identify specific drivers of disease progression may allow the future development of adjunctive targeted therapies in the setting of acquired resistance.

#### 4.1.1. Acquired Resistance to First Generation TKIs

Secondary EGFR mutations are the predominant mechanism of acquired resistance to first- and second-generation TKIs. Furthermore, the T790M mutation is solely attributed to 60% of acquired resistance cases with a presentation of indolent disease progression [97,135,182,183,184,185]. The mutation alters the ATP binding cleft preventing the binding of first- and second-generation TKIs [186]. Disease progression occurs with the positive selection of resistant clones indicated by an increasing proportion of circulating tumour cells expressing the resistant allele across serial measurements [187]. Circulating T790M cells did not indicate the onset of resistance but were a prognostic factor for earlier disease progression on first-generation TKIs. T790M not only arises during treatment but has also been identified in treatment-naive tumours and as a germline mutation inducing familial lung cancer predisposition [111,188,189,190,191]. Evidence of pre-existing mutations is consistent with a direct oncogenic role and in vitro demonstrations of enhanced EGFR phosphorylation and cancer-cell survival [192]. However, T790M resistance is complex and can be modified by concurrent mutations such as L858R, while gefitinib binding was unaffected by the dual T790M/L858R tumour yet resistance in this case emerged due to increased ATP affinity in the setting of the L858R variant [193,194]. Resistance has also been identified in other mutations, such as T854A and L747S/P, which sterically hinder TKI binding [131,133,140,141].

The T790M variant prompted the development of further TKI generations to address treatment resistance. While second-generation TKIs were unable to inhibit T790M-EGFR in vivo at clinical concentrations despite promising preclinical data, the third-generation agent osimertinib was successful at achieving inhibition [150,195,196]. Osimertinib was effective as a rescue medication after failing first-generation TKIs, with a median PFS of 10.1 months compared to 4.4 months on platinum-based chemotherapy, and as a first-line therapy with a median PFS of 18.9 months compared to 10.2 months for first-generation TKIs [197,198]. However, progression remains inevitable with patients demonstrating resistance to third-generation TKIs and disease progression.

The appearance of secondary mutations or the loss of the T790M clone that occurs with the emergence of acquired resistance can guide further targeted therapy. For resistance that emerges with de novo L474P or G724S mutations, recent reports demonstrate the clinical efficacy of second-generation afatinib rescue therapy. The L474P mutation in the N-terminal of the βchain results in hydrophobic-centre conformational changes and constitutive kinase activation that was managed with afatinib with a much improved PFS of 24 months at the time of publication [140]. Similarly, the G724S mutation within the glycine-rich loop of the kinase domain induces a conformation change that prevents osimertinib-binding yet demonstrated in vitro sensitivity to afatinib [149,199]. This mutation was associated with T790M loss and an allelic bias towards 19del but not L858R tumours [149]. At the L817 residue, L718Q is the predominant mutation that confers steric hindrance to osimertinib-binding as a first- and second-line medication [200,201]. In as many as 50% of L718Q cases, a concurrent T790M mutation is lost at the time of disease progression, conferring sensitivity to first- and second-generation TKIs [200]. Hence, the status of the T790 residue may guide ongoing therapy at the time of disease progression.

#### 4.1.2. Acquired Resistance to Third-Generation TKIs

The emergence of EGFR-mutant clones that display clinical resistance to all current TKI generations indicate the need for further drug development. Specifically, G796 residue mutations display clinical resistance to all TKIs via steric hindrance, even with the loss of a concurrent cis T790M mutation in the resistant clone at the time of disease progression [145,146]. Similarly, the L792F/H mutations present with multiallelic resistance patterns that complicate targeted therapy. Specifically, the mutant residue induces resistance via steric hindrance of a methoxy group on the methyl ring of osimertinib, which prevents binding [202]. While sole L792 mutants demonstrate in vitro sensitivity to first-generation TKIs, the mutant often presents with multiple mutations that lead to pan-TKI resistance. In particular, the L792 mutation reduces kinase hydrophobicity that results in a concurrent L858R residue displaying first-generation TKI resistance with or without coexisting T790M [145,150,202].

The C797S mutation is a variant with no current treatment options, yet novel inhibitors demonstrate a proof-of-concept for future mutation-specific targeted therapy. Resistance to third generation TKIs is mediated by impaired osimertinib binding to the C797 residue within the ATP-binding cleft [143]. When the C797 variants emerge in trans with T790M and 19del, a combination of first- and third-generation TKIs achieved a PFS of 7.4 months until the emergence of a cis clone with pan-TKI resistance [143,203,204]. However, a novel preclinical allosteric inhibitor was found to inhibit EGFR kinase activity by targeting the inactive EGF monomer at an allosteric site outside the kinase binding cleft in the presence of the cetuximab-induced inhibition of dimerisation [144,205]. Achieving kinase inhibition outside the ATP-binding cleft that is targeted by current TKI therapies demonstrates a proof-of-concept for future therapies to manage and reduce the emergence of resistance.

#### 4.1.3. Acquired Resistance via Downstream Activation

Resistance to targeted kinase therapy emerges through signalling aberrations downstream of the initiating EGFR-mutation. These signalling aberrations emerge in post-treatment tumour biopsies and are attributed to resistance to all TKI generations via activation of mitogen-activated protein kinase kinase (MEK)/extracellular signal regulated kinase (ERK), and Akt/mTOR pathways. Within the Akt/mTOR pathway, mutations have been demonstrated in patient-derived tumours within PIK3CA, PTEN, and AKT1 that lead to increased Akt phosphorylation, mTOR-mediated survival signalling, and early disease progression [162,163,164,165]. Treatment with the mTOR inhibitor everolimus achieved a disappointing PFS of 2.1 months, indicating the requirement for targeted therapies in emergent resistance [165]. However, strategies are complicated by concurrent EGFR mutations, downstream mutations, and cross-activation from PIK3CA to MEK pathways [165,206]. Within the MEK/ERK proliferation pathway, BRAF and KRAS mutations contribute to resistance. BRAF mutations comprised 50% of V600E, whereas NRAF mutations were associated with an upstream KRAS mutation [207]. In vitro, resistance was overcome by MEK and BRAF inhibition and was dependent on ERK signalling with no AKT phosphorylation [166,167]. KRAS mutations were previously thought to be mutually exclusive with EGFR mutations and are a well-defined resistance mechanism to EGFR-mAbs in colorectal cancer [208,209]. However, post-TKI tumour biopsies have confirmed KRAS mutations in new osimertinib resistance in conjunction with new EGFR mutations, with resistance ameliorated via Ras-ERK inhibition in vitro [210,211]. Hence, mutations downstream of EGFR can induce or complicate the emergence of TKI resistance.

#### 4.1.4. Acquired Resistance via Alternative Pathways

Enhancement of alternative pathways is demonstrated to induce resistance to targeted kinase therapy by bypassing EGFR to activate MEK/ERK and AKT/mTOR signalling. MET signalling is a well-described pathway in which amplification or protein overactivation phosphorylates ErbB3 and bypasses EGFR to activate Akt [212]. MET amplification is associated with poorer patient outcomes and acquired resistance, including a median PFS of 3.5 months compared to 9.9 months without MET amplification. The MET copy number was a biomarker that predicted poor outcomes and poor response to therapy [213,214]. However, combination treatment with the MET/C-ros oncogene 1(ROS1)/anaplastic lymphoma kinase (ALK) inhibitor capmatinib and gefitinib in acquired resistance cases achieved a modest improvement of PFS to 5.5 months [215]. Resistance is proposed to emerge either from the selection of clones present in treatment-naïve tumours or through MET activation following the increased secretion of the endogenous ligand hepatocyte growth factor (HGF) [216,217]. Treatment options are limited for tumours that possess bypass signalling. For example, HER2 mutant or amplified tumours with bypass ERK activation achieved a mere 18% response rate and 3.9 months median PFS with trastuzumab/afatinib combination therapy [159,218]. Furthermore, the screening of patients with progressive disease has identified poorly described resistance-associated mutations involving multiple mechanisms. Resistance mediated via fibroblast growth factor receptor 1 (FGFR1) and MAPK mutations occurred through increased canonical ERK and ALK signalling [153,219,220]. However, mutations affecting the Crk-like protein (CRKL) and insulin-like growth factor 1 receptor (IGF1R) acted at the receptor complex to enhance downstream signalling via increased adaptor protein amplification or affinity, respectively [151,221,222]. Therefore, targeted therapy for bypass signalling in the setting of acquired TKI resistance is a developing field with ongoing current clinical trials.

#### 4.1.5. Acquired Resistance via Other Mechanisms

The heterogeneity of non-small cell lung cancer is evident in the diverse genetic and cellular mechanisms of acquired resistance. Histologic transformation represents the progression of a primary or secondary tumour to either a small cell or squamous cell carcinoma [223]. The transformed tumours are identifiable by possessing the same sensitising or resistance markers as the initial tumour yet adopting a clinical presentation and treatment sensitivity specific to the new histological classification [170]. Transformation occurs most commonly within the selection pressure of initial TKI treatment that either results in the expansion of a specific clone or the differentiation of cancer stem cells within the heterogenous tumour [170,174]. Epithelial to mesenchymal transition (EMT) can accompany transformation or occur in isolation from it and is associated with an upregulation of AXL and vimentin and a downregulation of E-cadherin, yet the significance has not yet been incorporated into clinical approaches [169]. Genetic mechanisms include the rare event of oncogene fusion, in which oncogenes activate downstream signalling and bypass EGFR inhibition [180]. Fusions are observed at a greater frequency in acquired resistance than in first-generation TKIs (16% vs. 3%) [177]. The study of ALK and RAF suggest that the specific binding partner of oncogene fusions may influence the emergence and drug-specificity of resistance amidst an increasing number of observed fusion partners [224,225]. Fusion-dependent tumours retained sensitising mutations and lost T790M resistance mutation in 75% of cases [177]. Acquired resistance is further attributed to novel mechanisms including epigenetic silencing via microRNA and nuclear factor kappa-light-chain-enhancer of activated B cells (NF-κB) transcriptional regulation in vitro, however, the significance has yet to be established in the clinic [175,176].

### 4.2. Monoclonal Antibody (mAb) Therapy

Therapeutic mAbs are used in the treatment of cancers by binding to the specific target cell antigens responsible for growth and differentiation. Anti-EGFR mAbs bind to the surface of EGFR via an antigen-binding fragment (Fab) and competitively block the binding of EGF [226]. Antibody-receptor complexes are then internalised and degraded, leading to EGFR down-regulation on the surface of tumour cells. Therapeutic mAbs also act via immulogical mechanisms such as antibody-dependent cellular cytotoxicity (ADCC), which involve antibodies coating the target cell, effector cells recognising the antibody, and consequent effector-cell-induced apoptosis [226].

Unfortunately, despite their success in other cancer types, such as colorectal carcinoma and head and neck SCC (Table 2), mAb therapy has had uninspiring results in NSCLC and is not currently approved for its treatment. Two phase III studies, FLEX and BMS099, have investigated the combination of cetuximab with chemotherapy in advanced NSCLC and demonstrated a minimal improvement and no improvement in overall survival, respectively [93,227]. Preclinical evidence demonstrates that mAbs can achieve disease control in dimerisation-dependent L858R tumours but not in dimerisation-independent 19del tumours [228]. This dimerisation-dependent mechanism underlies the clinical use of mAbs in EGFR-mutants that possess changes in the intermolecular dimerisation affinity. Specifically, structural modelling predicts that exon 20 insertions bias the electrostatic affinity towards the formation of active EGFR dimers through conformational changes that prevent C-helix reorientation to the inactive state [229]. The addition of cetuximab in combination with osimertinib or afatinib for the treatment of exon 20 insertion tumours have achieved a PFS of 5.4 months and 6.4 months, respectively [230]. Furthermore, osimertinib with necitumumab (another anti-EGFR mAb) combination therapy achieved a median PFS of 6.4 months in a tumour containing the EGFR C797S variant, which is predicted to have increased intermolecular interaction due to hydrogen bonding between the mutation and EGFR R841 [231,232]. Therefore, a greater structural understanding of receptor activation in EGFR-mutant tumours may yield biomarkers for mAb combination therapy in cases of acquired resistance.

## 5. Conclusions

EGFR-targeting therapies have dramatically altered the treatment landscape of a number of cancers. The underlying mechanism of EGFR overexpression has a significant impact on treatment response and the development of resistance. In cancers such as head and neck, where the majority of tumours overexpress EGFR, antibodies inducing effects such as ADCP and ADCC are clinically relevant. In NSCLC, a therapeutic approach is differentiated with regard to mutant-EGFR and the development of resistance to anti-EGFR therapy through various mechanisms including gene line polymorphisms, secondary EGFR mutations, enhancement of alternate signalling pathways, and downstream signalling pathway protein mutations. Further research into these mechanisms of resistance and the nature of EGFR expression will allow the development of superior therapeutic techniques to help improve the outcomes of patients with NSCLC. The approach to EGFR targeting in each cancer thus depends on the type of EGFR dysregulation specific to each tumour type.

## 6. Patents

Granted patents: PAT-02100-JP-01; 2015-538212; PAT-02100-GB-01; 13848409.2; PAT-02100-DE-01; 60 2013059561.5; PAT-02100-AU-02; 2013334493; PAT-02100-US-01 14/438440; PAT-02100-JP-01 2015-538212.

## Figures and Tables

**Table 1:** Comparison of the incidence of EGFR-activating mutations and EGFR overexpression amongst common cancers.

Cancer type	Frequency of Activating Mutations of EGFR	Study	Frequency of EGFR Overexpression	Sample Size	Method of Detection	Study
NSCLC	50% of Asian patients 10–15% of Caucasian patients	[33]	43–89%	96–515	IHC ^a^	[34,35,36,37,38,39,40,41,42,43]
			31–58%	2972	Meta-analysis	[44]
Colorectal carcinoma (CRC)	Rare–2.33%	[45]	51–75.5%	99–193	IHC	[46,47,48,49]
Head and neck SCC ^b^	Rare–1.72%	[45]	45%	115	IHC	[50]
			92%	24	Southern blot hybridisation	[51]
Pancreatic adenocarcinoma	Rare–0.78%	[45]	49–69%	32–181	IHC	[52,53,54]
Breast cancer	Rare–1.31%	[45]	27%	21,418	Meta-analysis	[55]
Prostate	Rare–0.82%	[45]	31–100%	74–98	IHC	[56,57]
Oesophageal SCC	Rare–2.72%	[45]	53.6–65%	56–152	IHC	[58,59]
Gastric	Rare–2.2%	[45]	27–44%	82–511	IHC	[60,61]
Hepatocellular carcinoma	Rare–1.59%	[45]	47–68%	53–100	IHC	[62,63]
Glioblastoma	17.56%	[45]	63%	49	IHC	[64]
Ovary	Rare–0.98%	[45]	28–33%	80	IHC	[65]
Bladder	Rare–3.28%	[45]	26.2–71%	72–126	IHC	[66,67]
Renal cell carcinoma	Rare–1.16%	[45]	21–98%	50–175	IHC	[68,69,70]

^a^ Immunohistochemistry (IHC), ^b^ squamous cell carcinoma (SCC).

**Table 2:** Currently approved anti-EGFR therapies and their indications.

Cancer Type	Tyrosine Kinase Inhibitors	Monoclonal Antibodies
NSCLC	1st generation Gefitinib-metastatic NSCLC with EGFR exon 19 deletion or exon 21 mutation (L858R) Erlotinib-metastatic or locally advanced NSCLC with EGFR exon 19 deletion or exon 21 mutation (L858R) 2nd generation Dacomitinib-metastatic NSCLC with EGFR exon 19 deletion or exon 21 mutation (L858R) Afatinib-metastatic NSCLC with EGFR exon 19 deletion or exon 21 mutation (L858R) 3rd generation Osimertinib-metastatic EGFR T790M mutation-positive NSCLC, with progressive disease following first-line EGFR TKI therapy Olmutinib-second-line treatment of NSCLC with T790M mutation in EGFR	
Pancreatic cancer	Erlotinib-metastatic or advanced pancreatic cancer in combination with gemcitabine	
Breast cancer	Neratinib-HER2-overexpressing breast cancer Lapatinib-HER2-overexpressing breast cancer	
Thyroid cancer	Vandetanib-medullary thyroid carcinoma	
CRC		Cetuximab-metastatic KRAS-negative CRC; in combination with chemotherapy or as a single agent Panitumumab-metastatic KRAS-negative CRC; in combination with chemotherapy or as a monotherapy in patients who have failed chemotherapy
Head and neck- SCC		Cetuximab-in combination with radiation therapy for locally advanced disease or in combination with chemotherapy for recurrent/metastatic disease

**Table 3:** Mechanisms of NSCLC resistance to TKI Therapy *.

Effector	Prevalence	Resistance To	Mechanism
Germ Line Polymorphisms			
EGFR-T790M	Preclinical [116]	1st Gen. TKIs	Allosteric hindrance of ATP-binding pocket, increased ATP affinity
EGFR-V843I	3/5 carriers developed disease [117]	1st Gen. TKIs	Steric hindrance, associated with additional L858R mutation [118,119,120]
BIM	Deletion in 12.9% of East Asian individuals [121]	1st/2nd/3rd TKIs	High BIM expression correlates with tumour apoptosis and enhanced PFS/OS [122,123,124,125,126,127]
Secondary EGFR Mutations			
S768I	9/1527 cases	1st Gen. TKIs	Attenuated BIM to reduce apoptosis, often with concurrent G719 or L858R [123,128]
D761Y	1/16 cases	1st Gen. TKIs	Reduced EGFR phosphorylation with additional L858R mutation [129,130]
T854A	1/48 cases	1st Gen. TKIs	Steric hindrance [131]
L747S	12/3648 [132]	1st Gen. TKIs	Steric hindrance [133]
T790M	98/155 cases [134]	1st/2nd Gen. TKI	Allosteric hindrance of ATP-binding pocket, increased ATP affinity [135]
Exon20 Insertion	Asia: 67/218 [136], 23/186 Europe [137]	1st/2nd/3rd Gen. TKIs	Conformational change inducing constitutive activation [136,137,138,139]
L747P	Case Report	1st/3rd Gen. TKIs	Conformational change inducing constitutive activation [140,141]
T790M Amplification	Preclinical	2nd Gen. TKIs	Reversible selection of amplified clone in response to TKI [142]
C797S	6/15 cases [143]	3rd Gen. TKIs	Mutation in EGFR prevents osimertinib binding, 84% co-occur with multiple resistance mechanisms [144]
G796S/R/D	23/93 cases	1st/3rd Gen. TKIs	Steric hindrance [145,146]
L792F/H	10/93 cases	3rd Gen. TKIs	Steric hindrance, arise in trans with T790M and cis with C797S [145,147]
L718Q	9/93 cases [145,148]	3rd Gen. TKIs	Steric hindrance [145]
G724S	4/30 cases	3rd Gen. TKIs	A glycine-rich loop conformation prevents initial reversible TKI binding. The mutation is associated with T790M loss, mutually exclusive to C979S, and afatinib-sensitive [149,150].
Enhancement of Alternate Pathways			
CRKL	1/11 cases [151]	1st/2nd Gen. TKIs	Amplification, leading to downstream activation of ERK and Akt [151,152]
MAPK	Case report	1st Gen. TKIs	ERK overexpression [153]
IGF1R	Preclinical	1st/2nd Gen. TKIs	Constitutive activation of PI3K/Akt pathway [154,155]
MET	4/18 1st Gen-resistant cases [156], 3/5 3rd Gen-resistant cases [157]	1st/3rd Gen. TKIs [156,157,158]	HER3-dependent PI3K activation [156]
HER2	3/26 cases [159], 2/5 3rd Gen-resistant cases [157]	1st/2nd/3rd Gen. TKIs [157,159]	Alternative receptor amplification, mutually exclusive with the T790M mutation [159]
FGFR1	1/23 cases [160]	3rd Gen. TKIs	Autocrine loop signalling [161]
Downstream Mutations			
PTEN	1/24 cases	1st/2nd Gen. TKIs	PI3K/Akt activation via PIK3CA [162]
PIK3CA	5/43 cases [163]	1st/2nd/3rd Gen. TKIs	PI3K/Akt activation [164]
AKT1	3/49 cases	1st/2nd/3rd Gen. TKIs	mTOR activation [165]
BRAF	2/195 cases	3rd Gen. TKIs	MEK and ERK overexpression [166,167]
KRAS	3/43 cases [167], 9/38 adenocarcinoma cases [168]	3rd Gen. TKIs	MAPK overexpression, mutually exclusive with the EGFR mutations [167,169,170]
EMT			
AXL (and ligand GAS6)	7/35 cases	1st/2nd/3rd Gen. TKIs	EMT with vimentin overexpression [169]
Histologic Transformation			
Chronic EGFR Inhibition	5/37 1st Gen-resistance cases [170], 2 3rd Gen-resistant case studies [171]	1st/3rd Gen. TKIs	Transformation to SCLC with EMT and potential retinoblastoma signalling [159,172,173]
Chronic EGFR Inhibition	1st Gen-resistant case study [172,173], 5/71 3rd Gen-resistant cases [174]	1st/3rd Gen. TKIs	Transformation from adenocarcinoma to SCC [172,173,174]
Transcriptional Regulation			
NF-κB	Statistical significance across 52 cases, Preclinical	1st Gen. TKIs	High NF-κB activity predicted resistance via survival signalling [175]
Epigenetics	Preclinical	1st Gen. TKIs	miR-21 expression reducing PTEN and PDCD4 activity [176]
Oncogene Fusion			
EIF4G2	1/32	1st/2nd Gen. TKIs	Treatment-resistance associated fusion to GAB1 [177]
ALK	108/2835	2nd/3rd Gen. TKI	Treatment-resistance associated fusion to EML4, STRN, CEBPZ predominantly in adenocarcinoma cases in minimal smokers [178,179]
NTRK1	4/3875 cases	2nd/3rd Gen. TKIs	Treatment-resistance associated fusion to LRRC71, PLEKHA6, RRL8, RP11 [178]
BRAF	1/31 with KIF5A [177], 10/3595 [180]	2nd/3rd Gen. TKIs	Treatment-resistance associated fusion to AGAP3, AGK, ARMC10, DOCK4, EPS15, KIAA1549, SALL2, TRIM24 [180]
RET	6/3875 cases	3rd Gen. TKIs	Treatment-resistance associated fusion to CCDC6, MCPA4, CDC123, KIF5B [178,181]
ROS1	3/3875 cases	3rd Gen. TKIs	Treatment-resistance associated fusion to DCBLD1 [178]
FGFR3	5/32	3rd Gen. TKIs	Treatment-resistance associated fusion to TACC3 [177]

* Please refer to Abbreviations for a list of the acronyms used in this table.

## Abbreviations

Acronym	Definition
AGAP3	ArfGAP with GTPase Domain, Ankyrin Repeat and PH Domain 3
AGK	Acylglycerol kinase
Akt	Protein kinase B
AKT1	AKT Serine/Threonine Kinase 1
ALK	Anaplastic lymphoma kinase
ARMC10	Armadillo Repeat Containing 10
ATP	Adenosine triphosphate
BIM	B cell lymphoma-2-like
BRAF	B-Raf Proto-Oncogene
CCDC6	Coiled-Coil Domain Containing 6
CDC123	Cell Division Cycle 123
CEBPZ	CCAAT Enhancer Binding Protein Zeta
CRKL	CRK like proto-oncogene
DCBLD1	Discoidin, CUB and LCCL Domain Containing 1
DOCK4	Dedicator of cytokinesis 4
EGFR	Epidermal growth factor receptor
EIF4G2	Eukaryotic translation initiation factor 4 gamma 2
EMT	Epithelial to mesenchymal transition
EPS15	Epidermal growth factor receptor substrate 15
ERK	Extracellular-signal-regulated kinase
FGFR	Fibroblast growth factor receptor
GAB1	Grb2 associated binding protein 1
GAS6	Growth arrest specific 6
HER	Human epidermal growth factor receptor 2
IGF1R	Insulin like growth factor 1 receptor
KIF5B	Kinesin Family Member 5B
KRAS	Kirsten rate sarcoma viral oncogene
LRRC71	Leucine rich repeat containing 71
MAPK	Mitogen-activated protein kinase
MEK	Mitogen-activated protein kinase kinase
miR-21	mircoRNA 21
mTOR	Mechanistic target of rapamycin
NF-κB	Nuclear factor kappa-light-chain-enhancer of activated B cells
NTRK1	Neurotrophic Receptor Tyrosine Kinase 1
PDCD4	Programmed Cell Death 4
PFS	Progression free survival
PI3K	Phosphoinositide 3-kinase
PI3KCA	PI3K catalytic subunit alpha
PLEKHA6	Pleckstrin homology domain containing A6
PTEN	Phosphatase and tensin homolog
OS	Overall survival
RET	Rearranged during transfection
ROS1	C-ros oncogene 1
SALL2	Spalt like transcription factor 2
SCC	Squamous cell carcinoma
SCLC	Small cell lung cancer
STRN	Striatin
TACC3	Transforming acidic coiled-coil containing protein 3
TKI	Tyrosine kinase inhibitor
TRIM24	Tripartite Motif Containing 24
